# Magnetic Charge Model for Leakage Signals from Surface Defects in Ferromagnetic Material

**DOI:** 10.3390/ma16103750

**Published:** 2023-05-15

**Authors:** Xinyu Li, Guangming Sheng, Zimin Meng, Fan Qin, Zhifeng Liu

**Affiliations:** School of Mechanical Engineering, Hefei University of Technology, Hefei 230009, China; shenggm2022@163.com (G.S.); mengzimin1027@163.com (Z.M.); peak.liu@263.net (Z.L.)

**Keywords:** magnetic dipole, round hole defects, metal magnetic memory, numerical integration

## Abstract

A novel three-dimensional theoretical model of magnetic flux leakage (MFL) is proposed in this paper based on the magnetic dipole model. The magnetic dipole model assumes that a ferromagnetic specimen with defects is exposed to a uniform external magnetic field that causes a uniform magnetization around the defect surface. Under this assumption, the MFL can be regarded as arising from magnetic charges on the defect surface. Previous theoretical models were mostly used to analyze simple crack defects such as cylindrical and rectangular cracks. In this paper, we developed a magnetic dipole model for more complex defect shapes such as circular truncated holes, conical holes, elliptical holes, and double-curve-shaped crack holes to complement the existing defect shapes. Experimental results and comparisons with previous models demonstrate that the proposed model provides a better approximation of complex defect shapes.

## 1. Introduction

Non-destructive testing technology is used to detect stress defects in many industries, especially in important industrial fields such as energy, automotive, shipping, and aerospace. Among them, metal magnetic memory testing is a widely used, non-destructive testing technology for ferromagnetic components. Leakage detection technology exposes the specimen to a constant size and known direction magnetic field, while metal magnetic memory testing technology utilizes the earth’s magnetic field. When there is a crack defect on the surface, there is a leakage of magnetic flux near the defecting surface. Therefore, the leakage magnetic field generated by the defect contains important shape information regarding the surface damage defect. Therefore, the geometric shape of surface fracture defects in ferromagnetic materials can be accurately evaluated by metal magnetic memory testing [1]. Therefore, metal magnetic memory testing can be used in places where conventional detection methods such as rails, pipelines, and pressure vessels are difficult to detect. Shi associated magnetic dipole theory with stress and established a force–magnetic coupling magnetic dipole theory, which allows the magnetic dipole model to analyze the effects of various influencing factors on leakage magnetic field signals. Based on the rectangular groove defect, he also provided the first analytical expression for the magnetic dipole of trapezoidal groove defects, further expanding the form of the defect leakage magnetic field signal [2,3]. Mandache, Taniguchi, and Suresh analyzed the leakage magnetic field signals of cylindrical defects, associated them with defect size, and experimentally verified the effectiveness of the model [4,5,6]. Trevino et al., established an improved analytical magnetic dipole model to represent the 3D magnetic leakage field caused by surface-breaking defects on ferromagnetic specimens and verified the accuracy of the improved magnetic dipole model using finite element simulations [7]. Shi described a magnetic charge model for metal magnetic memory signals and simulated specimens with stress concentration zones and long elliptical defects based on this model [8]. Okolo et al., simulated the distribution characteristics of surface and far-field leakage fields using axial magnetization technology and detected and characterized the leakage distribution caused by surface and far-field hairline cracks on rectangular specimens [9]. Leng and Han separately established magnetic dipole integral models to describe the leakage fields generated by the plastic zone at the tip of a V-shaped notch in ferromagnetic materials due to dislocation accumulation and magnetic dipole models to evaluate the stress concentration caused by local plastic deformation [10,11]. Wu et al., analyzed the variation in surface magnetic charge density for defects in different directions and effectively described the directional influence of defects on the distribution of leakage magnetic field [12,13]. Li et al., proposed a “near-field” magnetic leakage method to quantify defect width, indicating that the distance between the two peaks of the “near-field” leakage field is closely related to the defect width and lift-off value, but not to the defect depth [14]. Xu et al., used the equivalent magnetic charge method to establish a self-leakage magnetic model for buried defects, which successfully evaluated the characteristics of buried defects [15]. He et al., established a mathematical model of magnetic field signals, providing theoretical and experimental evidence to identify the stress state of pipeline circumferential welds [16]. Suresh et al., proposed an analytical model for predicting the leakage magnetic field signals of surface defects in ferromagnetic pipes, which can be used for the rapid prediction of leakage magnetic field signals and for inputting data regarding defect reconstruction into leakage inverse problems [17]. Yang et al., derived and established a specific mathematical model of low-frequency magnetic leakage field based on the magnetic dipole theory, considering four types of crack defects with an equal length and width but different bottom shapes [18]. Liu et al., developed a numerical model for detecting axial cracks in pipelines based on electromagnetic theory and calculated the electromagnetic detection signals of cracks of different sizes and orientations [19]. Long et al., discussed the corresponding relationship between the defect leakage field signal and the defect opening contour model based on the fundamental principles of electromagnetic fields and developed an approximate method to detect the defect edge [20].

Although many experts and scholars have conducted extensive research and analyses of rectangular groove-type cracks on metal surfaces and have obtained the expression of the surface crack leakage magnetic field signal, there are not many analyses of various circular hole defects on the surface. Based on the magnetic dipole model of rectangular and cylindrical crack defects, this paper obtains the integral expression of the leakage magnetic defect signals of conical holes, elliptical holes, hyperbolic crack holes, and truncated cone holes, and verifies the rationality of the expression through experiments, providing a new method for quantitatively analyzing the variation in leakage magnetic signals on the surface of defects. At the same time, it also provides a new analysis method for difficult-to-detect locations and regions, such as stress corrosion defects with elliptical shapes in boilers and saddle-shaped defects in reactor pressure vessels [21,22].

## 2. Rectangular Magnetic Dipole Theoretical Model

**Figure 1:**
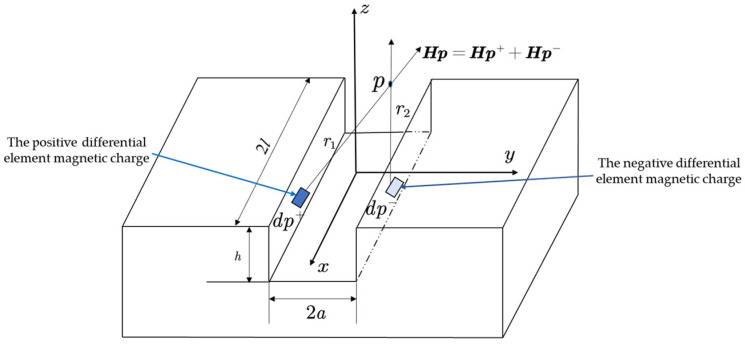
Rectangular groove defective magnetic dipole model.

Based on the theory of magnetic dipole for cylindrical hole defects in the literature [3], it is believed that the presence of defects on the surface of ferromagnetic materials will generate positive and negative opposite magnetic charges at the defect wall, which, in turn, will generate a leakage magnetic field on the defect surface. Taking the rectangular groove crack defect as an example, combined with the magnetic dipole model analysis, as shown in Figure 1, where *l* denotes the length of the crack, *d* denotes the width of the crack, and *h* the depth of the crack, there exists a magnetic dipole band with equal surface magnetic charge density on both sides of the rectangular groove, and a pair of equal positive and negative differential element magnetic charges *dp^+^* and *dp^−^* are taken on the face of the rectangular groove crack defect, where *r*_1_ and *r*_2_ are the distances from the positive and negative magnetic charges to point *p*, respectively. The magnitudes of the magnetic fields generated at point *p* are ${dH}_{p}^{+}$ and ${dH}_{p}^{-}$, their expressions are given by Equations (1) and (2), respectively, so that the total magnetic field *dH_p_* generated by the equivalent magnetic dipole at point *p* is the sum of ${dH}_{p}^{+}$ and ${dH}_{p}^{-}$. Meanwhile, the micro-magnetic charge can be expressed by the product of surface magnetic charge density and area, i.e., *dp*, and *dp* can be expressed by Equation (3), where $\mu_{0}$ is the vacuum permeability, *σ*_s_ is the magnetic charge density of the surface, and *ds* is the area of the micro-element in which the magnetic charge is located. Based on the analytical model of magnetic dipole for rectangular groove defects, the foundation for the establishment of magnetic dipole model for cylindrical hole-type defects is laid.

(1) $$dH_{p}^{+}=\frac{dp^{+}}{4\pi \mu_{0}r_{1}^{3}}\vec{r_{1}}$$

(2) $$dH_{p}^{+}=\frac{dp^{-}}{4\pi \mu_{0}r_{2}^{3}}\vec{r_{2}}$$

(3) $$dp=\sigma_{s}ds$$

## 3. Modeling of Cracking Defects

### 3.1. Cylindrical Hole-Shaped Defect

**Figure 2:**
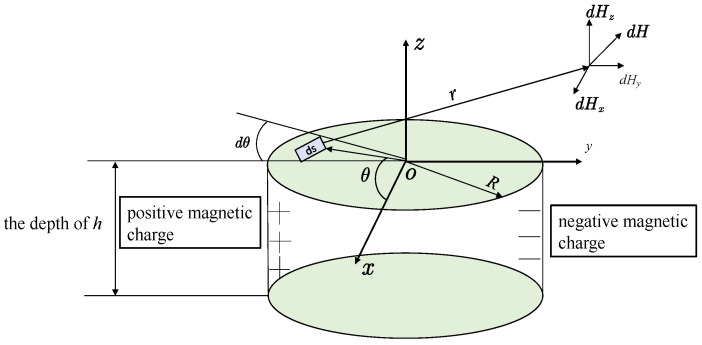
Cylindrical hole-shaped defective magnetic dipole model.

Assuming that a positive and negative equal surface magnetic charge also exists on the surface of the cylindrical hole defect, the interaction between the ambient magnetic field and the surface cracked defect leads to a spontaneous leakage magnetic field on the defect surface of the ferromagnetic material, which forms a magnetic charge distribution on its defect surface similar to the form of a uniform charge distribution. Consider a cylindrical hole-shaped defect, whose model schematic is shown in Figure 2, assuming that the cylindrical hole defect surface is filled by the surrounding air domain and embedded in the ferromagnetic medium. The cylindrical hole-shaped defect is divided into two parts, the left part with a positive magnetic charge and the right part with a negative magnetic charge; both positive and negative magnetic charges are uniformly distributed around the inner surface of the cylindrical hole-shaped defect. The main surface parameters of the cylindrical hole defect are the radius of the defect is *R*; the depth of the defect is *h*; the distance of any differential element magnetic charge on the defect surface to the point *p* is *r*. The top center point of the cylindrical defect is selected as the coordinate origin; the *z*-axis is perpendicular to the upper surface of the cylinder and passes the coordinate origin; the top region of the cylindrical defect is centered at (0, 0, 0) and the bottom region is centered at (0, 0, −*h*). Since the magnetic memory probe is measured in the region of *z* > 0 during the actual signal measurement, the analysis focuses on the leakage magnetic field signal generated in the region of *z* > 0 for the type of cylindrical hole defect. *dp* for the differential element magnetic charge on the defect surface, and *ds* for the differential element area selected for the surface magnetic charge, which is expressed in column coordinates as *ds*, with du being the length of the differential element area along the axis direction. *dp* is still expressed by Equation (3) and *ds* is expressed by Equation (4). Since the cylinder is symmetric at both the *xoz* plane and the *yoz* plane, the defect spontaneous leakage magnetic field signal along the *x*-axis and along the *y*-axis is practically the same. For the convenience of analysis, it is assumed that the positive and negative magnetic charges are symmetrically and uniformly distributed about the *xoz* plane. The defect surface with a positive magnetic charge is taken as an example to analyze the leakage magnetic field signal at the measurement point *p*. Assuming that the measurement trajectory is measured along the *y*-axis, the coordinates of the measurement point *p* are (0, *y*, *z*) and the coordinates of the positive magnetic charge differential element are (−*Rcosθ*, −*Rsinθ*, *u*); then, the distance *r*_1_ from the positive magnetic charge differential element to the measurement point can be obtained, where $r_{1}^{z}$ denotes the component of vector $\vec{r_{1}}$ along the *z*-axis direction $r_{1}^{z}$ = *z* − *u*, $r_{1}^{y}$ denotes the component of vector $\vec{r_{1}}$ along the *y*-axis direction; that is, *r* = *y* + *Rsinθ*. The distance *r*_2_ from the negative magnetic charge differential element to the measurement point can be similarly obtained; the *z*-axis vector component *r* = *z − u*, and the *y*-axis vector component is *r* = *y* − *Rsinθ*, so the differential element of positive and negative magnetic charge surface to the differential element of leakage magnetic field signal at the measurement point *p* can be expressed by Equation (5). Equations (5) and (6) can be integrated, and the corresponding integral expressions can be summed up to obtain Equation (7), which provides the integral expressions for the total tangential and normal directions of the cylindrical hole-shaped defect at measurement point *p*.

(4) ds=Rdθdu

(5) $$\left\{\begin{array}{c} dH_{z}^{+}=\frac{dp^{+}}{4\pi \mu_{0}r_{1}^{2}}r_{1}^{z}=\frac{\sigma_{s}R}{4\pi \mu_{0}r_{1}^{2}}r_{1}^{z}d\theta du\\ dH_{z}^{-}=-\frac{dp^{+}}{4\pi \mu_{0}r_{2}^{2}}r_{2}^{z}=-\frac{\sigma_{s}R}{4\pi \mu_{0}r_{2}^{2}}r_{2}^{z}d\theta du\\ dH_{y}^{+}=\frac{dp^{+}}{4\pi \mu_{0}r_{1}^{2}}r_{1}^{y}=\frac{\sigma_{s}R}{4\pi \mu_{0}r_{1}^{2}}r_{1}^{y}d\theta du\\ dH_{y}^{-}=-\frac{dp^{+}}{4\pi \mu_{0}r_{2}^{2}}r_{2}^{y}=-\frac{\sigma_{s}R}{4\pi \mu_{0}r_{2}^{2}}r_{2}^{y}d\theta du \end{array}\right.$$

(6) $$\left\{\begin{array}{c} H_{z}^{+}=\frac{\sigma_{s}R}{4\pi \mu_{0}}\int_{0}^{\pi }d\theta \int_{-h}^{0}\frac{\left(z-u\right)}{{\left[{\left(R\cos\theta \right)}^{2}+{\left(y+R\sin\theta \right)}^{2}+{\left(z-u\right)}^{2}\right]}^{\frac{3}{2}}}du\\ H_{z}^{-}=-\frac{\sigma_{s}R}{4\pi \mu_{0}}\int_{0}^{\pi }d\theta \int_{-h}^{0}\frac{\left(z-u\right)}{{\left[{\left(R\cos\theta \right)}^{2}+{\left(y-R\sin\theta \right)}^{2}+{\left(z-u\right)}^{2}\right]}^{\frac{3}{2}}}du\\ H_{y}^{+}=\frac{\sigma_{s}R}{4\pi \mu_{0}}\int_{0}^{\pi }d\theta \int_{-h}^{0}\frac{\left(y+R\sin\theta \right)}{{\left[{\left(R\cos\theta \right)}^{2}+{\left(y+R\sin\theta \right)}^{2}+{\left(z-u\right)}^{2}\right]}^{\frac{3}{2}}}du\\ H_{y}^{-}=-\frac{\sigma_{s}R}{4\pi \mu_{0}}\int_{0}^{\pi }d\theta \int_{-h}^{0}\frac{\left(y-R\sin\theta \right)}{{\left[{\left(R\cos\theta \right)}^{2}+{\left(y-R\sin\theta \right)}^{2}+{\left(z-u\right)}^{2}\right]}^{\frac{3}{2}}}du \end{array}\right.$$

(7) $$\left\{\begin{array}{c} H_{z}=H_{z}^{+}+H_{z}^{-}\\ H_{y}=H_{y}^{+}+H_{y}^{-} \end{array}\right.$$

### 3.2. Circular Truncated Hole Defect

**Figure 3:**
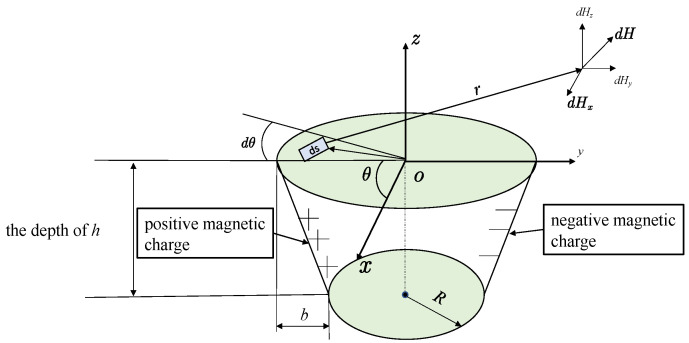
Circular truncated hole defective magnetic dipole model.

Because the radius *R* of the cylindrical defective hole does not change with depth, while the radius *R* of the cylindrical surface of the circular truncated hole defect is changed linearly with depth, $R^{\prime }=R+b+ub/h$, *R*′ is the radius of the defective hole at depth *u*. The depth *h* of the circular truncated hole is shown in Figure 3; therefore, the difference in the size of the depth *h* of the circular truncated hole will also have a different effect on the defect leakage magnetic field signal. On the basis of the cylindrical hole defect, the *R* in its integral expression is replaced by *R*′ to obtain the integral expression of the tangential and directional leakage magnetic field signal of the circular truncated hole defect at measurement point *p*. This is expressed by Equation (8).

(8) $$\left\{\begin{array}{c} H_{z}^{+}=\frac{\sigma_{s}}{4\pi \mu_{0}}\int_{0}^{\pi }d\theta \int_{-h}^{0}\begin{array}{c} \left(R+b+ub/h\right)\cdot \end{array}\\ \frac{\left(z-u\right)}{{\left[{\left(\left(R+b+ub/h\right)\cos\theta \right)}^{2}+{\left(y+\left(R+b+ub/h\right)\cos\theta \right)}^{2}+{\left(z-u\right)}^{2}\right]}^{\frac{3}{2}}}du\\ H_{z}^{-}=-\frac{\sigma_{s}}{4\pi \mu_{0}}\int_{0}^{\pi }d\theta \int_{-h}^{0}\left(R+b+ub/h\right)\cdot \\ \frac{\left(z-u\right)}{{\left[{\left(\left(R+b+ub/h\right)\cos\theta \right)}^{2}+{\left(y-\left(R+b+ub/h\right)\sin\theta \right)}^{2}+{\left(z-u\right)}^{2}\right]}^{\frac{3}{2}}}du\\ H_{y}^{+}=\frac{\sigma_{s}}{4\pi \mu_{0}}\int_{0}^{\pi }d\theta \int_{-h}^{0}\left(R+b+ub/h\right)\cdot \\ \frac{\left[y+\left(R+b+ub/h\right)\sin\theta \right]}{{\left[{\left(\left(R+b+ub/h\right)\cos\theta \right)}^{2}+{\left(y+\left(R+b+ub/h\right)\sin\theta \right)}^{2}+{\left(z-u\right)}^{2}\right]}^{\frac{3}{2}}}du\\ H_{y}^{-}=-\frac{\sigma_{s}}{4\pi \mu_{0}}\int_{0}^{\pi }d\theta \int_{-h}^{0}\left(R+b+ub/h\right)\cdot \\ \frac{\left[y-\left(R+b+ub/h\right)\sin\theta \right]}{{\left[{\left(\left(R+b+ub/h\right)\cos\theta \right)}^{2}+{\left(y-\left(R+b+ub/h\right)\sin\theta \right)}^{2}+{\left(z-u\right)}^{2}\right]}^{\frac{3}{2}}}du \end{array}\right.$$

### 3.3. Conical Hole-Shaped Defect

**Figure 4:**
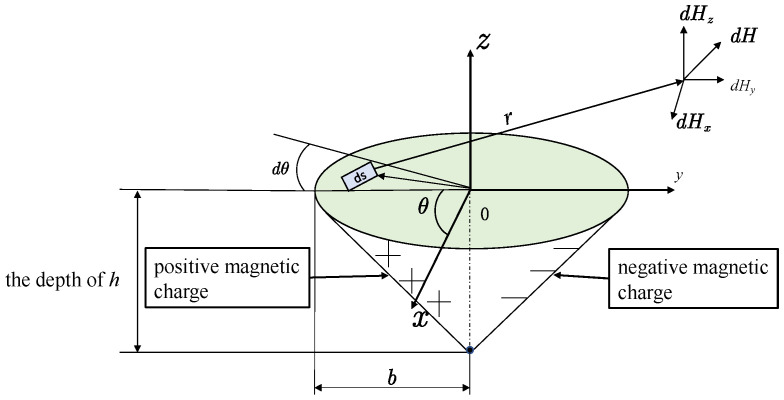
Conical hole-shaped defective magnetic dipole model.

When *R* = 0, the circular truncated hole defect can be transformed into a conical hole-shaped defect, as shown in Figure 4, so that the integrated expression of the circular truncated hole defect leakage magnetic field signal *R* = 0 can be obtained as conical hole-shaped defect leakage magnetic field signal integral expression (9).

(9) $$\left\{\begin{array}{c} H_{z}^{+}=\frac{\sigma_{s}}{4\pi \mu_{0}}\int_{0}^{\pi }d\theta \int_{-h}^{0}\left(b+ub/h\right)\cdot \\ \frac{\left(z-u\right)}{{\left[{\left(\left(b+ub/h\right)\cos\theta \right)}^{2}+{\left(y+\left(b+ub/h\right)\cos\theta \right)}^{2}+{\left(z-u\right)}^{2}\right]}^{\frac{3}{2}}}du\\ H_{z}^{-}=-\frac{\sigma_{s}}{4\pi \mu_{0}}\int_{0}^{\pi }d\theta \int_{-h}^{0}\left(b+ub/h\right)\cdot \\ \frac{\left(z-u\right)}{{\left[{\left(\left(b+ub/h\right)\cos\theta \right)}^{2}+{\left(y-\left(b+ub/h\right)\sin\theta \right)}^{2}+{\left(z-u\right)}^{2}\right]}^{\frac{3}{2}}}du\\ H_{y}^{+}=\frac{\sigma_{s}}{4\pi \mu_{0}}\int_{0}^{\pi }d\theta \int_{-h}^{0}\left(b+ub/h\right)\cdot \\ \frac{\left(y+\left(b+ub/h\right)\sin\theta \right)}{{\left[{\left(\left(b+ub/h\right)\cos\theta \right)}^{2}+{\left(y+\left(b+ub/h\right)\sin\theta \right)}^{2}+{\left(z-u\right)}^{2}\right]}^{\frac{3}{2}}}du\\ H_{y}^{-}=-\frac{\sigma_{s}}{4\pi \mu_{0}}\int_{0}^{\pi }d\theta \int_{-h}^{0}\left(b+ub/h\right)\cdot \\ \frac{\left(y-\left(b+ub/h\right)\sin\theta \right)}{{\left[{\left(\left(b+ub/h\right)\cos\theta \right)}^{2}+{\left(y-\left(b+ub/h\right)\sin\theta \right)}^{2}+{\left(z-u\right)}^{2}\right]}^{\frac{3}{2}}}du \end{array}\right.$$

### 3.4. Elliptical Hole-Shaped Defect

**Figure 5:**
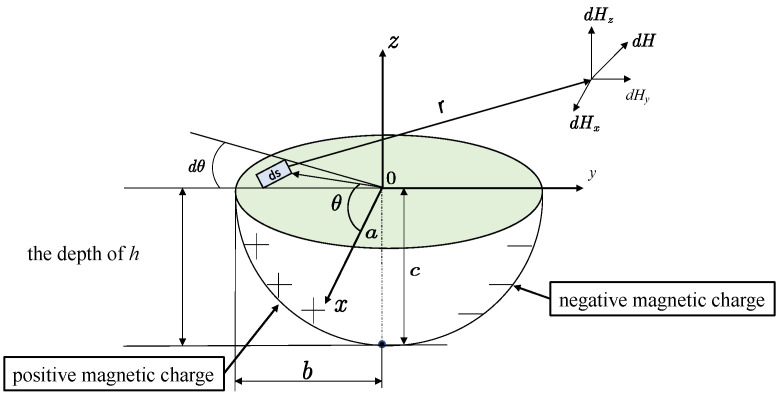
Elliptical hole-shaped defective magnetic dipole model.

The established magnetic dipole model of the elliptical hole-shaped defect is shown in Figure 5. Converting the right-angle coordinate system into a spherical coordinate system, the angle between the magnetic charge differential element and the *x*-axis is *θ*, and the angle between the magnetic charge differential element and the *z*-axis is *φ*. Since the general ellipsoidal formula is $x^{2}/a^{2}{+y}^{2}/a^{2}+u^{2}/c^{2}=1$, the following formula can be obtained by converting it into a spherical coordinate system.

(10) $$\left\{\begin{array}{c} x_{0}=a\cos\theta \sin\varphi \\ y_{0}=b\sin\theta \sin\varphi \\ u_{0}=c\cos\varphi \end{array}\right.$$

The expression of the magnetic charge differential element *dp* = *σ*_s_*R*^2^sin*θdθdz* when *R* = *R_t_* is:

(11) $$R_{t}=\sqrt{x_{0}^{2}+y_{0}^{2}+u_{0}^{2}}=\sqrt{{\left(a\cos\theta \sin\varphi \right)}^{2}+{\left(b\sin\theta \sin\varphi \right)}^{2}+{\left(c\cos\varphi \right)}^{2}}$$

In turn, the expression for the magnetic charge differential element in the spherical coordinate system is obtained as:

(12) $$dp=\sigma_{s}ds=\sigma_{s}R_{t}^{2}\sin\theta d\theta d\varphi =\sigma_{s}\left[{\left(a\cos\theta \sin\varphi \right)}^{2}+{\left(b\sin\theta \sin\varphi \right)}^{2}+{\left(c\sin\varphi \right)}^{2}\right]\sin\theta d\theta d\varphi$$

The position of the magnetic charge differential element on the elliptical surface is:

(13) $$\left\{\begin{array}{c} \begin{array}{c} x=R_{t}\cos\theta \sin\varphi \\ y=R_{t}\sin\theta \cos\varphi \end{array}\\ u=R_{t}\cos\varphi \end{array}\right.$$

In turn, the integral expression of the leakage field signal in the tangential and normal directions for elliptical hole-type defects is obtained as:

(14) $$\left\{\begin{array}{c} H_{z}^{+}=\frac{\sigma_{s}}{4\pi \mu_{0}}\int_{0}^{\pi }d\theta \int_{\frac{\pi }{2}}^{\pi }\frac{R_{t}^{2}\sin\varphi \cdot \left(z-R_{t}\cos\varphi \right)}{{\left[{\left(R_{t}\cos\theta \sin\varphi \right)}^{2}+{\left(y+R_{t}\sin\theta \sin\varphi \right)}^{2}+{\left(z-R_{t}\cos\varphi \right)}^{2}\right]}^{\frac{3}{2}}}\sin\theta d\varphi d\theta \\ H_{z}^{-}=-\frac{\sigma_{s}}{4\pi \mu_{0}}\int_{0}^{\pi }d\theta \int_{\frac{\pi }{2}}^{\pi }\frac{R_{t}^{2}\sin\varphi \cdot \left(z-R_{t}\cos\varphi \right)}{{\left[{\left(R_{t}\cos\theta \sin\varphi \right)}^{2}+{\left(y-R_{t}\sin\theta \sin\varphi \right)}^{2}+{\left(z-R_{t}\cos\varphi \right)}^{2}\right]}^{\frac{3}{2}}}\sin\theta d\varphi d\theta \\ H_{y}^{+}=\frac{\sigma_{s}}{4\pi \mu_{0}}\int_{0}^{\pi }d\theta \int_{\frac{\pi }{2}}^{\pi }\frac{R_{t}^{2}\sin\varphi \cdot \left(y+R_{t}\sin\theta \sin\varphi \right)}{{\left[{\left(R_{t}\cos\theta \sin\varphi \right)}^{2}+{\left(y+R_{t}\sin\theta \sin\varphi \right)}^{2}+{\left(z-R_{t}\cos\varphi \right)}^{2}\right]}^{\frac{3}{2}}}\sin\theta d\varphi d\theta \\ H_{y}^{-}=-\frac{\sigma_{s}}{4\pi \mu_{0}}\int_{0}^{\pi }d\theta \int_{\frac{\pi }{2}}^{\pi }\frac{R_{t}^{2}\sin\varphi \cdot \left(y-R_{t}\sin\theta \sin\varphi \right)}{{\left[{\left(R_{t}\cos\theta \sin\varphi \right)}^{2}+{\left(y-R_{t}\sin\theta \sin\varphi \right)}^{2}+{\left(z-R_{t}\cos\varphi \right)}^{2}\right]}^{\frac{3}{2}}}\sin\theta d\varphi d\theta \end{array}\right.$$

When *a* = *b* = *c* = *R*, Equation (14) is the same as the equation of the magnetic dipole model of the spherical hole in the literature [23], which verifies the reasonableness of the change in the model equation from the side.

### 3.5. Double-Curve-Shaped Crack Hole Defect

**Figure 6:**
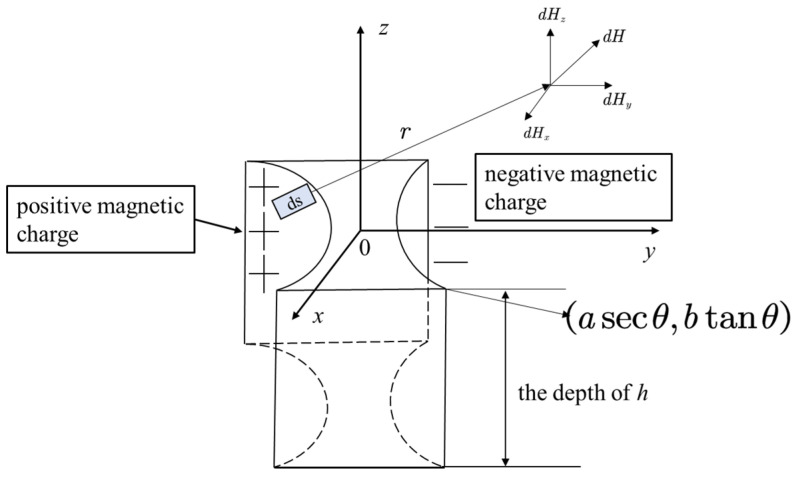
Double-curve-shaped crack hole defective magnetic dipole model.

The established magnetic dipole model of the double-curve-shaped crack hole defect is shown in Figure 6. The right-angle coordinate system is transformed into a column coordinate system. The formula for the hyperbola on the *xoy* plane is $y^{2}/a^{2}{\;-\;x}^{2}/b^{2}=1$, and the parametric coordinates of the hyperbola are *x = btanθ*, *y = asecθ*; the magnetic charge area differential element *ds = dldu*, where *dl* can be expressed by Equation (11). Then, the integral expression of the leakage magnetic field signal in the form of the hyperbola cracked hole is obtained, as shown in Equation (16).

(15) $$dl=\sqrt{{\left(dx\right)}^{2}+{\left(dy\right)}^{2}}=\sqrt{b^{2}\sec^{4}\theta +a^{2}\sec^{2}\theta \tan^{2}\theta }d\theta$$

(16) $$\begin{array}{l} H_{z}^{+}=\frac{\sigma_{s}}{4\pi }\int_{-h}^{0}du\int_{-a\sec\theta }^{a\sec\theta }\frac{\left(z-u\right)\sqrt{b^{2}\sec^{4}\theta +a^{2}\sec^{2}\theta \tan^{2}\theta }}{{\left[{\left(b\tan\theta \right)}^{2}+{\left(y+a\sec\theta \right)}^{2}+{\left(z-u\right)}^{2}\right]}^{\frac{3}{2}}}d\theta \\ H_{z}^{-}=\frac{\sigma_{s}}{4\pi }\int_{-h}^{0}du\int_{-a\sec\theta }^{a\sec\theta }\frac{\left(z-u\right)\sqrt{b^{2}\sec^{4}\theta +a^{2}\sec^{2}\theta \tan^{2}\theta }}{{\left[{\left(b\tan\theta \right)}^{2}+{\left(y-a\sec\theta \right)}^{2}+{\left(z-u\right)}^{2}\right]}^{\frac{3}{2}}}d\theta \\ H_{y}^{+}=\frac{\sigma_{s}}{4\pi }\int_{-h}^{0}du\int_{-a\sec\theta }^{a\sec\theta }\frac{\left(y+a\sec\theta \right)\sqrt{b^{2}\sec^{4}\theta +a^{2}\sec^{2}\theta \tan^{2}\theta }}{{\left[{\left(b\tan\theta \right)}^{2}+{\left(y+a\sec\theta \right)}^{2}+{\left(z-u\right)}^{2}\right]}^{\frac{3}{2}}}d\theta \\ H_{y}^{-}=\frac{\sigma_{s}}{4\pi }\int_{-h}^{0}du\int_{-a\sec\theta }^{a\sec\theta }\frac{\left(y-a\sec\theta \right)\sqrt{b^{2}\sec^{4}\theta +a^{2}\sec^{2}\theta \tan^{2}\theta }}{{\left[{\left(b\tan\theta \right)}^{2}+{\left(y-a\sec\theta \right)}^{2}+{\left(z-u\right)}^{2}\right]}^{\frac{3}{2}}}d\theta \end{array}$$

## 4. Comparison and Validation of Theoretical Models

Since the model-derived dual integral $F=\int_{a}^{b}dx\int_{c}^{d}f(x,y)dy$ is non-integrable, combined with the binary complexified Simpson’s product formula, the integral region of the rectangle is divided equally into *n* × *m* small rectangles. Four vertices are taken in the middle of each small rectangle in equal parts, and the Simpson’s product formula for each small rectangle can be obtained by combining the four vertices.

$$F=\frac{\left(b-a\right)\left(d-c\right)}{36mn}\left\{f\left(x_{0},y_{0}\right)+f\left(x_{2},y_{0}\right)-f\left(x_{0},y_{2}\right)+f\left(x_{2},y_{2}\right)+\right.\;\left.4\left[f\left(x_{0},y_{1}\right)+f\left(x_{1},y_{0}\right)+f\left(x_{2},y_{1}\right)+f\left(x_{1},y_{2}\right)\right]+16f\left(x_{1},y_{1}\right)\right\}\;\;$$

After summing them, we can obtain:

$$\int_{a}^{b}dx\int_{c}^{d}f\left(x,y\right)dy\approx G$$

where the *G* equation is equal to:

$$G=\frac{\left(b-a\right)\left(d-c\right)}{36mn}\overset{m-1}{\underset{j=0}{\sum }}\overset{n-1}{\underset{i=0}{\sum }}\left\{f\left(x_{2i},y_{2j}\right)+f\left(x_{2i+2},y_{2j}\right)\right.+f\left(x_{2i+1},y_{2j+2}\right)+f\left(x_{2i},y_{2j+2}\right)\;+\left.4\left[f\left(x_{2i+1},y_{2j}\right)+f\left(x_{2i+2},y_{2j+1}\right)+f\left(x_{2i+1},y_{2j+2}\right)+f\left(x_{2i},y_{2j+1}\right)+16f\left(x_{2i+1},y_{2j+1}\right)\right]\right\}\;\;=\frac{\left(b-a\right)\left(d-c\right)}{36mn}\overset{2m}{\underset{j=0}{\sum }}\overset{2n}{\underset{i=0}{\sum }}\lambda_{ij}f\left(x_{i},y_{j}\right)\;\;\;\;\;\;\;\;\;\;\;\;\;\;\;\;\;\;\;$$

*λ_ij_* are the coefficients of the complexified Simpson product formula, and *λ_ij_* are the elements of the following λ-matrices.

$\lambda =\left[\begin{array}{cccccccccc} 1 & 4 & 2 & 4 & 2 & \cdots & 4 & 2 & 4 & 1\\ 4 & 16 & 8 & 16 & 8 & \cdots & 16 & 8 & 16 & 4\\ 2 & 8 & 4 & 8 & 4 & \cdots & 8 & 4 & 8 & 2\\ & & \cdots & & & \cdots & & & \cdots & \\ & & \cdots & & & \cdots & & & \cdots & \\ & & \cdots & & & \cdots & & & \cdots & \\ 4 & 16 & 8 & 16 & 8 & \cdots & 16 & 8 & 16 & 4\\ 2 & 8 & 4 & 8 & 4 & \cdots & 8 & 4 & 8 & 2\\ 4 & 16 & 8 & 16 & 8 & \cdots & 16 & 8 & 16 & 4\\ 1 & 4 & 2 & 4 & 2 & \cdots & 4 & 2 & 4 & 2 \end{array}\right]$ and the error of the complexified Simpson product formula is:

$$E_{r}=-\frac{\left(b-a\right)\left(d-c\right)}{180}\left[{\left(\frac{b-a}{2}\right)}^{4}\frac{\partial^{4}f\left(\alpha ,\beta \right)}{\partial x^{4}}+{\left(\frac{d-c}{2}\right)}^{4}\frac{\partial^{4}f\left(\bar{\alpha },\bar{\beta }\right)}{\partial y^{4}}\right]$$

where $\alpha ,\bar{\alpha }\in (a,b),\;\beta ,\bar{\beta }\in \left(c,d\right)$.

The original reference [3] expresses the rectangular slot defect as:

(17) $$H_{x}=\frac{\sigma_{s}}{2\pi \mu_{0}}\left[\arctan\frac{h+y}{a-x}+\arctan\frac{h+y}{a+x}-\arctan\frac{y}{a-x}-\arctan\frac{y}{a+x}\right]$$

(18) $$H_{y}=\frac{\sigma_{s}}{4\pi \mu_{0}}\ln\left[\frac{{\left(a+x\right)}^{2}+{\left(h+y\right)}^{2}}{{\left(a+x\right)}^{2}+y^{2}}\cdot \frac{{\left(a-x\right)}^{2}+y^{2}}{{\left(a-x\right)}^{2}+{\left(y+h\right)}^{2}}\right]$$

Take the magnetic charge density as *σ_s_* = 4*πμ*_0_*δ_s_*; that used to obtain rectangular groove defects; magnetic charge density is $\delta_{s}=\frac{2.65}{2\pi }\left(\frac{h/a+1}{h/\left(\mu a\right)+1}\right);$ the magnetic charge density of cylindrical hole-shaped defects is $\delta_{s}=\frac{2.65}{2\pi }\left(\frac{h/R+1}{h/\left(\mu R\right)+1}\right)H_{a}$; the magnetic charge density of the conical hole-shaped defect is $\delta_{s}=\frac{2.65}{2\pi }\left(\frac{h/\left(R+2b\right)+1}{h/\mu \left(R+2b\right)+1}\right)H_{a}$; the magnetic charge density of the elliptical hole-shaped defect is $\delta_{s}=\frac{2.65}{2\pi }\left(\frac{c/\left[\left(a+b\right)/2\right]+1}{c/\mu \left[\left(a+b\right)/2\right]+1}\right)H_{a},$ where $\mu_{0}$ is the vacuum magnetic permeability, $\mu_{0}{=4\pi \;\times \;10}^{-7},\;\;H_{a}=50$ A/m. 

**Figure 7:**
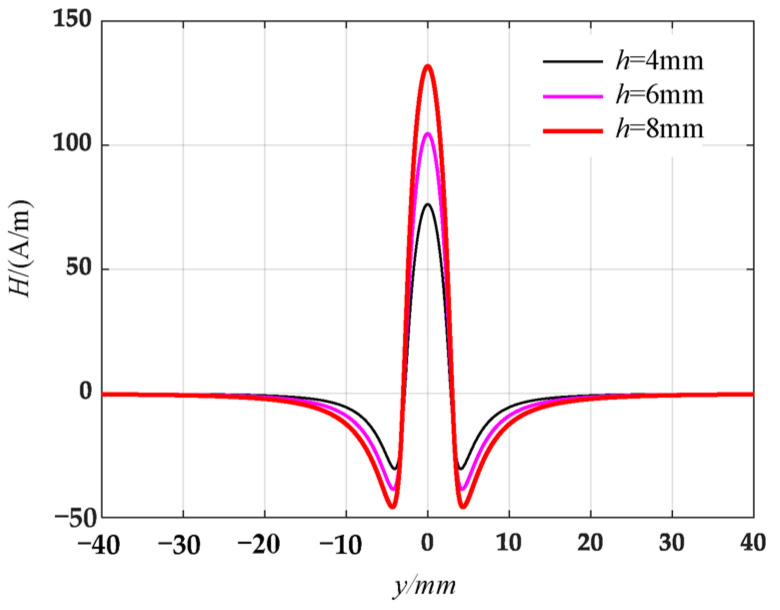
Distribution of tangential leakage field signals of circular truncated hole defects at different depths.

**Figure 8:**
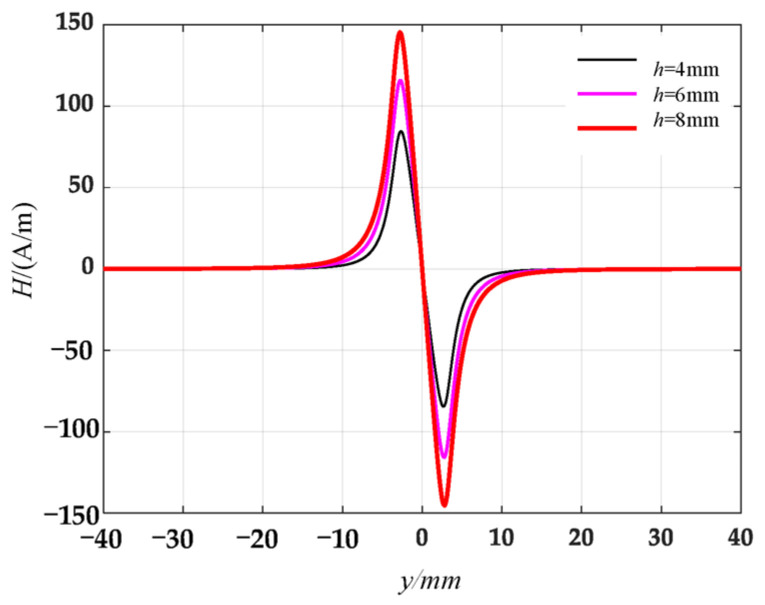
Distribution of normal leakage magnetic field signals of circular truncated hole defects at different depths.

At different defect depths, the tangential and normal leakage magnetic field signals are calculated by the circular truncated hole defective magnetic dipole model, and the results are shown in Figure 7 and Figure 8.

The analysis of circular truncated hole defects using the complexified Simpson product formula is similar to the results of the damage signal analysis of rectangular slot defects derived in the literature [3] and can thus expand the existing defects.

## 5. Materials and Methods

### 5.1. Metal Magnetic Memory Simulation

**Figure 9:**
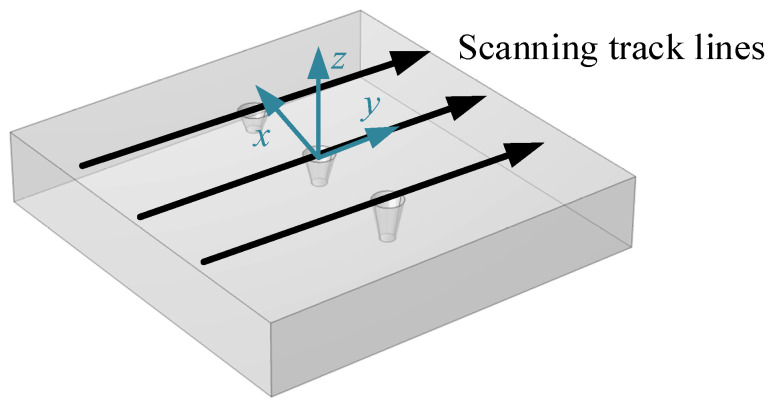
Three-dimensional model of circular truncated hole defects.

**Figure 10:**
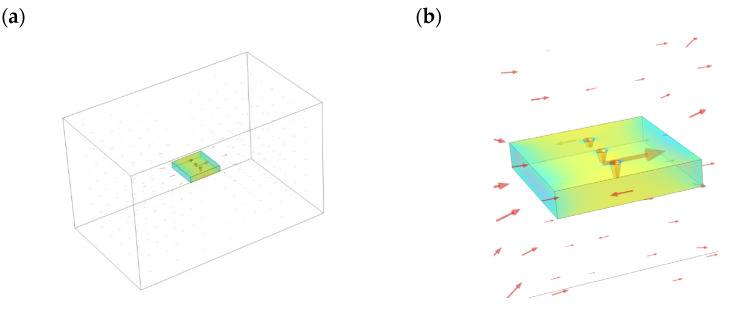
Schematic diagram of simulation results of metal magnetic memory: (**a**) full view of simulation; (**b**) specimen and surrounding magnetic field distribution map.

**Figure 11:**
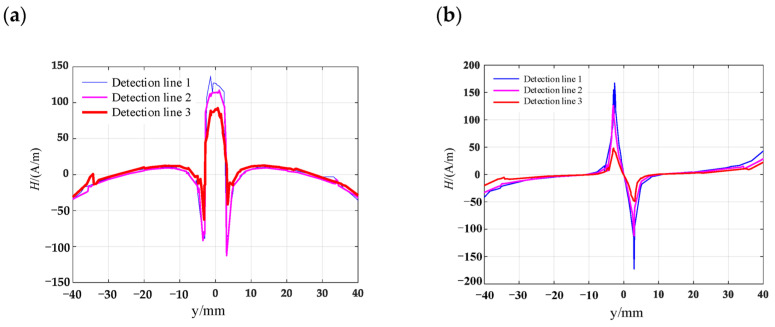
Schematic diagram of the simulation of magnetic field leakage signal from a circular truncated hole: (**a**) tangential leakage magnetic field signal; (**b**) normal leakage magnetic field signal.

A three-dimensional finite element model of the magnetic memory detection shown in Figure 9 is established, and the black arrow represents the scanning trajectory and direction. The simulation coordinate system selects the position in the middle of the surface of the three-dimensional model. The geometric model material is selected as 45-gauge steel, and the circular truncated hole defects with different depths are set on the surface, where the length and width of the material are 100 mm, 100 mm, and 15 mm, respectively. Select the scanning line close to the surface along the y-axis in the positive direction above the symmetrical center of each defect, and calculate the electromagnetic field using the magnetic field without current module, and the intrinsic relationship in the air domain satisfies ${B=\mu }_{0}H$. Since the magnetic memory detection is simulated in the geomagnetic field, the geomagnetic field used the length, width and height dimensions of 500 mm, 300 mm and 300 mm, respectively, and the magnetic field size is set to 50 A/m, with the magnetic field direction along the positive direction of the *y*-axis to make the model’s internal magnetic field strength uniform. The intrinsic relationship of the specimen material is calculated using the magnetization relationship of ${B=\mu }_{0}\left(H+M\right)$. Figure 10 shows a schematic diagram of the magnetic field simulation results of the circular truncated cone defect. The red arrow in the figure indicates the direction of the magnetic field at different positions in space. It can be seen that under the condition of uniform external magnetic field, ferromagnetic materials will undergo polarization. Its magnetization vector will start from one end and return to the other end, thus forming a complete loop. When there is a defect in the material, it causes a magnetic signal to be generated in the defect area, which increases the local magnetization strength and leads to local magnetic field distortion. Here, the leakage magnetic field signal on the surface directly above each circular truncated cone is scanned, and the results are obtained as shown in Figure 11. It is not difficult to see that the tangential component of the leakage magnetic field signal peaks at the cracked hole defect damage site, and the normal component of the leakage magnetic field signal crosses the zero point.

### 5.2. Experimental Verification and Analysis

The experimental material used in this study is common engineering structural steel 45, with the chemical composition listed in Table 1. The 45 steel was processed into rectangular plates with a thickness of 15 mm and dimensions of 100 mm by 100 mm. This type of steel has good strength and machinability and is often used in engineering for wear-resistant and high-toughness parts.

**Figure 12:**
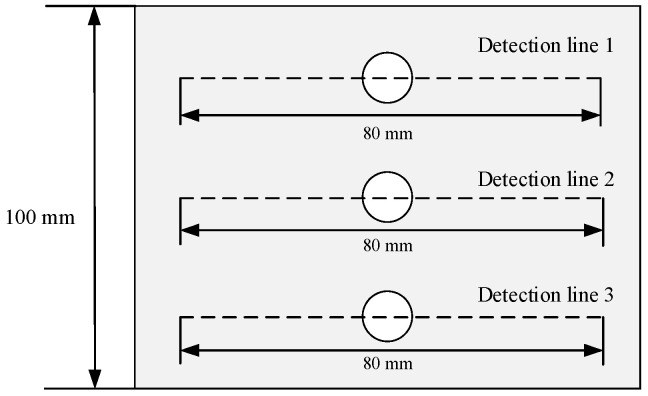
Schematic diagram of leakage magnetic field signal detection for the specimen.

**Figure 13:**
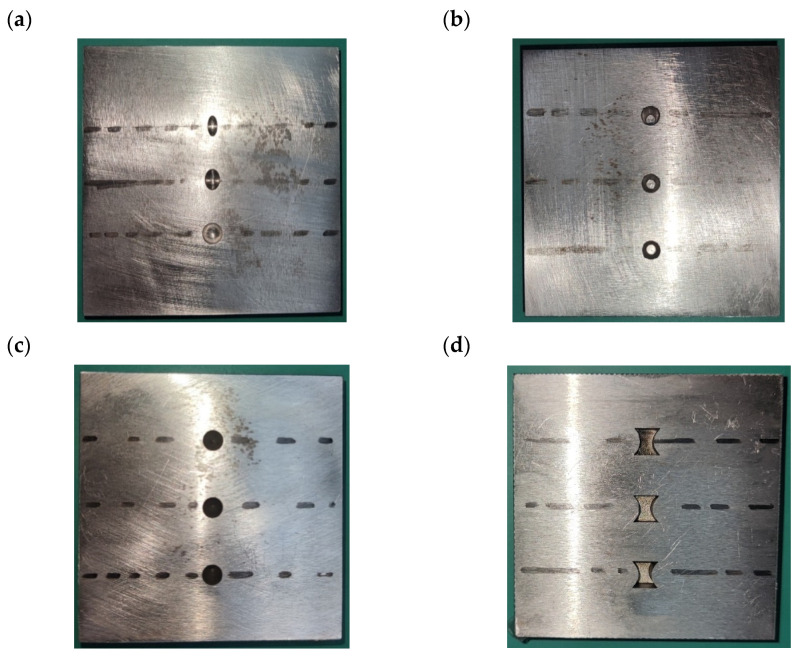
Processed defective specimens: (**a**) elliptical hole-shaped defect; (**b**) circular truncated hole-shaped hole defect; (**c**) conical hole-shaped defect; (**d**) double curve cracked hole defect.

**Figure 14:**
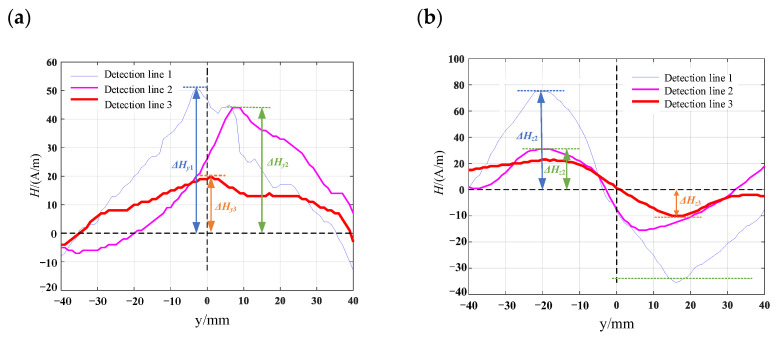
Leakage signal of elliptical hole-shaped defects: (**a**) tangential leakage magnetic field signal; (**b**) normal leakage magnetic field signal.

**Figure 15:**
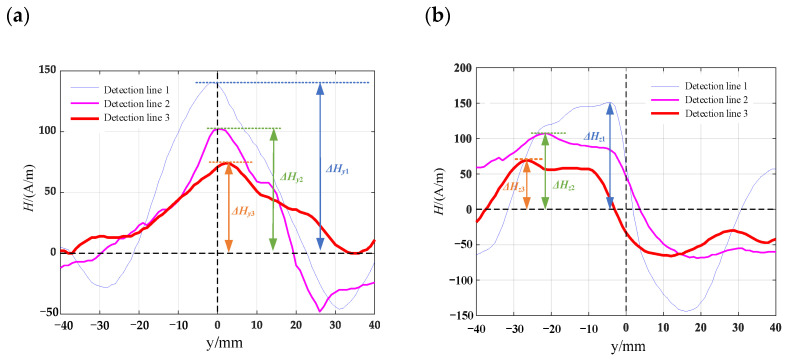
Leakage magnetic field signal of circular truncated hole defects: (**a**) tangential leakage magnetic field signal; (**b**) normal leakage magnetic field signal.

**Figure 16:**
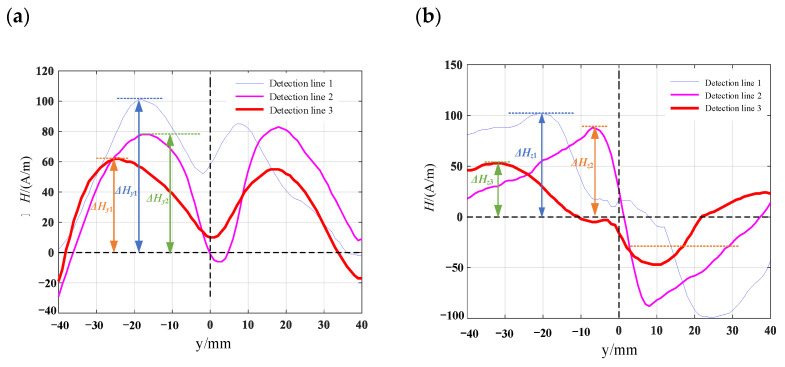
Leakage field signal of conical hole-shaped defects (**a**) tangential leakage magnetic field signal; (**b**) normal leakage magnetic field signal.

**Figure 17:**
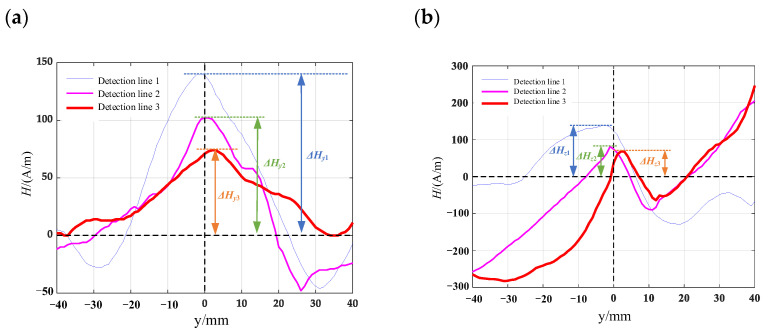
Leakage magnetic signal of double curved cracked hole defects: (**a**) tangential leakage magnetic field signal; (**b**) normal leakage magnetic field signal.

Computer numerical control machine tools are used to process various surface defect shapes on the surface of the material specimen. The pictures of the processed patterns are shown in Figure 12 and mark one detection line along the length direction of the specimen in the defect detection area. The length of the detection line is 80 mm. Using the TSC-2M-8 metal magnetic memory detector, the detection probes are measured along the detection lines on the specimen surface, and the detection line 1, detection line 2 and detection line 3 corresponded to the crack depths from the maximum to the minimum. Each specimen is prefabricated with three different defect depths of 8 mm, 6 mm, and 4 mm, respectively. To reduce the influence of signals between defects, the spacing between prefabricated defects is set to 25 mm. The prefabricated defect specimens are shown in Figure 13. The measurement results of the surface leakage magnetic field signal for defects are shown in Figure 14, Figure 15, Figure 16 and Figure 17. By analyzing the tangential component *H_y_* and normal component *H_z_* of the measured signal and combining the magnetic dipole model and spontaneous leakage magnetic theory, it can be concluded that the leakage magnetic field signal has a peak in the tangential component *H_y_* and a characteristic of passing through zero in the normal component *H_z_*. Moreover, the peak value on each detection line is different for each defect. Combining the magnetic dipole model and metal magnetic memory simulation results, it can be found that as the depth of defects increases, the amplitude of leakage magnetic field signals also increases. Therefore, it is possible to reflect the depth of defects with different shapes through leakage magnetic field signals, which is consistent with the expected results of the model.

**Figure 18:**
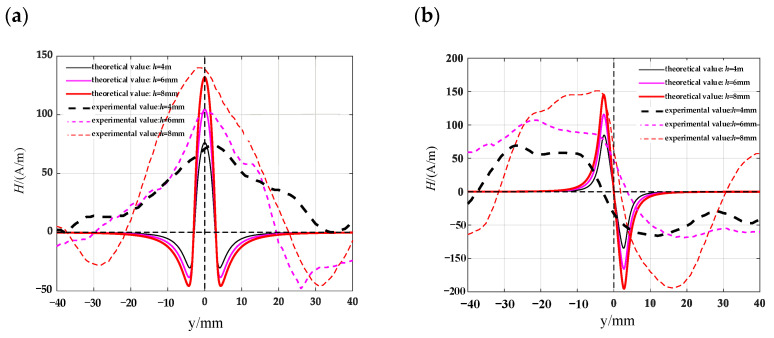

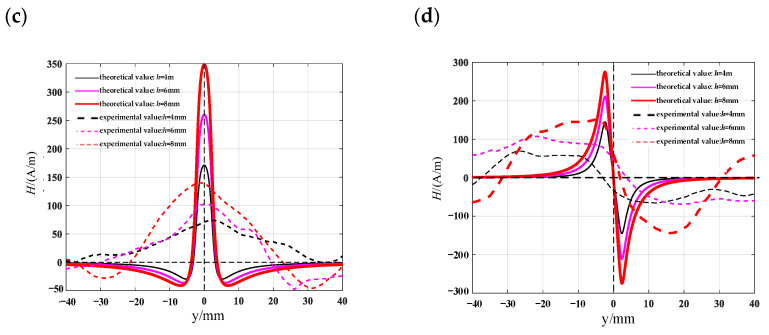
Comparison of theoretical and actual leakage magnetic field signals of defects. (**a**) comparison between the magnetic dipole model of a circular truncated hole and the actual tangential leakage magnetic field signal; (**b**) comparison between the magnetic dipole model of a circular truncated hole and the actual normal leakage magnetic field signal; (**c**) comparison between the rectangular crack model and the actual tangential leakage magnetic field signal; (**d**) comparison between the rectangular crack model and the actual normal leakage magnetic field signal.

Combining the measured magnetic leakage signals with the theoretical models of the given defect shape and the existing simple rectangular crack model, the established model can better express the magnetic leakage signals of complex-shaped defects. Taking the circular truncated hole as an example, the circular truncated hole defect is approximated by a rectangular crack form and compared with the actual signal. Meanwhile, the magnetic dipole model of the established circular truncated hole defect is also compared with the actual measured signal. A rectangular slot defect is used to replace the circular truncated hole. The half-width a of the rectangular slot is approximately represented by half of the sum of the upper and lower radii of the circular truncated hole. The upper radius of the circular truncated hole is 3 mm and the lower radius is 1.5 mm. The height of the lift-off value is selected to be 1 mm. The theoretical value of the magnetic flux leakage signal is calculated by substituting these values into the formula. The results are shown in Figure 18, where Figure 18a,b shows the tangential and normal signal comparison diagrams of the circular truncated hole defect theoretical model and the actual magnetic leakage field signal, respectively, and Figure 18c,d shows the tangential and normal signal comparison diagrams of the theoretical model of the rectangular crack defect approximation and the actual magnetic leakage field signal, respectively. It can be seen that the theoretical model of the circular truncated hole defect can better express the relationship between the depth and the peak value of the magnetic leakage field signal, indicating that the theoretical model of the simple rectangular crack is not accurate enough to express the complex magnetic leakage field signal and cannot accurately reflect the depth of the defect.

## 6. Results

Along the detection line direction, although the tangential and normal leakage magnetic signal values of each specimen are different, the change trend is roughly the same, and the tangential leakage magnetic signal of each defective specimen is approximately mirror-symmetric, and the normal leakage magnetic signal is approximately symmetrical about the origin. As shown in Figure 14, the peak value Δ*H_y_* of the tangential magnetic leakage signal of the elliptical hole increases with the depth of the defect; the peak value Δ*H_z_* of the normal magnetic leakage signal of the elliptical hole defect also increases with the depth of the defect, and the signal is symmetric at about the zero point. In fact, the increase in the width of the elliptical hole defect will cause the peak value of the magnetic leakage signal to decrease, but the effect of the depth of the defect on the signal is much greater than that of the width. As shown in Figure 15, the peak value Δ*H_y_* of the tangential magnetic leakage signal component of the circular truncated hole increases with the depth, and the curves of detection line 2 and detection line 3 are relatively close. The peak value Δ*H_z_* of the normal magnetic leakage signal component of the circular truncated hole defect also increases with depth. As shown in Figure 16, the peak value Δ*H_y_* of the tangential magnetic leakage signal component of the conical hole defect increases with the depth, and a low valley is found at the center position of the conical hole. The peak value Δ*H_z_* of the normal magnetic leakage signal component of the conical hole defect also increases with depth. As shown in Figure 17, the peak value Δ*H_y_* of the tangential magnetic leakage signal component of the double-curve-shaped crack hole defect increases with depth, and the peak value Δ*H_z_* of the normal magnetic leakage signal component near the zero point also increases with depth. The model established based on the magnetic dipole theory for the elliptical, circular truncated cone and double-curve-shaped crack holes agrees with the experimental results, but the tangential magnetic leakage signal of the conical hole defect does not completely agree, while the experimental results of the normal magnetic leakage signal still agree. Therefore, there are differences in the leakage signals of different types of defects, and further improvement of the theoretical model is needed in the future.

## 7. Conclusions

Based on the magnetic dipole theory model, the leakage magnetic field signals of different types of circular hole defects are physically modelled and the corresponding integral expressions are derived, which make a useful addition to the existing types of defects. The integral expressions of the leakage field signal for different types of circular hole defects are given, and the dual integrals are numerically computed by compounding Simpson’s formula. The leakage field signal images of the defects with different shapes are analyzed. The validity of the expression is experimentally verified.

## 8. Discussion

This paper mainly uses magnetic dipole theory to establish and analyze the magnetic flux leakage signals of complex defects without considering the deflection of defect direction, the defects located inside the material, and the influence of too close a defect spacing on the magnetic flux leakage signal. Since actual defects may deflect at different angles, it is impossible to ensure that the measurement path moves along the center of the defect symptoms during the measurement process. Therefore, it is necessary to consider the impact of defect deflection in different directions on magnetic flux leakage, and this can be solved by combining the coordinate transformation in reference [12]. For defects that exist inside the material and are a certain distance from the surface, this can be improved by combining the equivalent magnetic charge method in reference [15]. For multiple closely spaced defects, this can be analyzed by combining the superposition between multiple magnetic dipole models in reference [24]. If all these factors are taken into account, it will improve the application range of this paper’s theoretical model.

## Figures and Tables

**Table 1:** Chemical composition of experimental material 45# steel.

Element	C	Si	Mn	S	P
mass fraction	0.42~0.50%	0.17~0.37%	0.50~0.80%	≤0.035%	≤0.035%

## Data Availability

Data are available from the corresponding author upon reasonable request.
